# Asymmetric T-cell division: insights from cutting-edge experimental techniques and implications for immunotherapy

**DOI:** 10.3389/fimmu.2024.1301378

**Published:** 2024-03-01

**Authors:** Yaroslav Kaminskiy, Irina Ganeeva, Vitaly Chasov, Anna Kudriaeva, Emil Bulatov

**Affiliations:** ^1^ Department of Oncology and Pathology, Karolinska Institutet, SciLifeLab, Solna, Sweden; ^2^ Institute of Fundamental Medicine and Biology, Kazan Federal University, Kazan, Russia; ^3^ Shemyakin-Ovchinnikov Institute of Bioorganic Chemistry, Russian Academy of Sciences, Moscow, Russia

**Keywords:** T cell, T cell division, asymmetric division (AD), immunotherapy, T cell fate

## Abstract

Asymmetric cell division is a fundamental process conserved throughout evolution, employed by both prokaryotic and eukaryotic organisms. Its significance lies in its ability to govern cell fate and facilitate the generation of diverse cell types. Therefore, attaining a detailed mechanistic understanding of asymmetric cell division becomes essential for unraveling the complexities of cell fate determination in both healthy and pathological conditions. However, the role of asymmetric division in T-cell biology has only recently been unveiled. Here, we provide an overview of the T-cell asymmetric division field with the particular emphasis on experimental methods and models with the aim to guide the researchers in the selection of appropriate *in vitro*/*in vivo* models to study asymmetric division in T cells. We present a comprehensive investigation into the mechanisms governing the asymmetric division in various T-cell subsets underscoring the importance of the asymmetry in fate-determining factor segregation and transcriptional and epigenetic regulation. Furthermore, the intricate interplay of T-cell receptor signaling and the asymmetric division geometry are explored, shedding light on the spatial organization and the impact on cellular fate.

## Introduction

The factors determining cell fate depend largely on the course of division of the mother cell—symmetric or asymmetric. The cellular diversity is a fundamental requirement in developmental biology, and the asymmetric cell division is one of the mechanisms that create such a diversity.

Asymmetric division, which influences fate in many cell types, involves the establishment and maintenance of polarity during division, resulting in two daughter cells with different molecular compositions and fates (1). Asymmetric division controls the development and homeostasis of the brain, muscle, gut, mammary gland, and skin through the differential inheritance of fate determinants mediated by polarity proteins such as atypical protein kinase C (aPKC), discs large (Dlg), and Scribble (2, 3).

When a mother cell divides into two daughter cells with distinct fates, this is known as asymmetric division. Sometimes, the two daughters are identical when they are born, and the difference in their fates is discovered later, for example, through signaling from nearby cells (4). An alternative is to polarize the mother cell, in which case the two daughters are already distinct at birth. Most of these intrinsically asymmetric cell divisions follow a pattern of four steps. First, the mother cell’s symmetry is disrupted. Second, the mother cell also starts to become polarized. Third, fate determinants get concentrated in specific areas of the polarized mother cell. Fourth, the daughter cells’ distinct fates are achieved through the alignment of the mitotic spindle, which ensures that cleavage results in the proper partitioning of determinants to the daughter cells (2). These four procedures allow a mother cell to produce two daughter cells that are born simultaneously but are not identical. Specific cell types can be positioned at predetermined locations in a developing organism through asymmetric cell divisions, and a general idea for the orientation of such divisions is starting to take shape. In the mother cell, a polarity axis is first established and coordinated with the overall body plan. Then, cell-fate determinants are concentrated asymmetrically along this axis, and the mitotic spindle is oriented along this axis during mitosis, resulting in two daughter cells with differing concentrations of these determinants (5).

Asymmetric cell division supports the formation of cellular diversity in a wide variety of circumstances in eukaryotes, enabling uneven segregation of cell destiny determinants across sister cells during mitosis (1, 6–8). Through the asymmetric segregation of differentiation and aging factors, asymmetric division of budding yeast, *C. elegans* oocytes, Drosophila neuroblasts, and mouse neural stem cells, for example, helps to generate both self-renewing and differentiating progeny (7, 9–11).

Moreover, the importance of the endoplasmic reticulum (ER) in the asymmetric partition of a wide range of cellular components, ranging from protein aggregates, ubiquitinated proteins, and non-chromosomal DNA to transcription factors and other fate determinants, has been made demonstrated by studies of the mechanisms of asymmetric cell division in yeast, tissue culture cells, and mouse neural stem cells (12–15). In these cells, a barrier to lateral diffusion in the anticipated plane of cleavage compartmentalizes the ER membrane into two domains, one in each of the potential daughter cells. The split of aging factors and fate determinants is supported by these distinct areas. Such barriers have been observed in developing yeast cells, the nematode *C. elegans* one-celled embryo, and neural stem cells. The enrichment for long ceramides in the ER membrane at the barrier site is necessary for the creation of this barrier, at least in yeast. Because most ER membrane proteins’ transmembrane domains are tailored to the thinner bilayer that makes up the majority of the ER membrane, this causes the lipid bilayer to thicken locally and precludes the passage of most ER membrane proteins.

As discussed below, naïve CD8+ T cells divide and develop into short-lived effector cells (SLECs) that are important for immunological regulation and long-lived memory cells that are important for long-term immunity after being activated. The setting of activation and whether the activated cell divides symmetrically or asymmetrically have a significant impact on the percentage of memory cells that are produced.

This review begins with an examination of conserved asymmetric division mechanisms in T cells, shedding light on their crucial role in T-cell immunity regulation. Next, the intricate interplay of T-cell receptor (TCR) signaling in the asymmetric division is explored, emphasizing its significance in the asymmetric division incidence. Furthermore, the paper elucidates the role of asymmetric division in differentiated T-cell subsets, highlighting its diverse implications in immune responses. The asymmetry in T-cell fate-determining factors is also discussed followed by the transcriptional and epigenetic characterization of T-cell asymmetric division. Finally, the geometric aspects of T-cell asymmetric division are explored, illustrating the spatial organization and its impact on T-cell fate. We will not discuss cellular division mechanisms and direct interested readers to multiple excellent reviews of this topic (1, 16, 17).

## Where it all started

Chang et al. (2007) were the first to demonstrate asymmetric division phenomenon in T cells (18). They transferred transgenic P14 CD8+ T cells into C57BL/6 mice 24 h after infection with gp33 Listeria monocytogenes (LM-gp33). For the analysis of CD4+ T cells, naïve transgenic WT15 CD4+ T cells [carboxyfluorescein succinimidyl ester (CFSE)] were transferred into B10.D2 mice 24 h after Leishmania infection. Next, undivided cells (brightest CFSE) were sorted 32 h after transfer and fixed. The cells were analyzed by confocal microscopy in three configurations, namely, pre-mitotic cells [large with single microtubule-organizing center (MTOC)], mitotic cells (two MTOCs), and conjoined twin daughters. To assess the expression of specific proteins, the cells were stained with cluster of differentiation 4, cluster of differentiation 8, cluster of differentiation 90, Lymphocyte function-associated antigen 1, interferon-gamma receptor, Interleukin-2 receptor alpha chain, aPKC, Scribble, and tubulin.

The authors managed to show that the proximal daughter cell [daughter cell that remained attached to antigen-presenting cell (APC)] inherited most of CD3, CD4/8, LFA-1, IFNγR, and Scribble, whereas the distal daughter cell (daughter cell that budded away from APC) preferentially inherited aPKC. Moreover, CD62L, Interleukin-2 receptor alpha chain (IL-2Rα), and granzyme B (GZMB) exhibited a bimodal distribution in T cells that undergone first division. Importantly, without synapse formation with APCs, the division asymmetry was lost. To demonstrate the biological relevance, the first-division daughters (identified by the second brightest CFSE peak) were sorted into CD8^hi^ and CD8^lo^ populations after 48 h of LM infection. These populations were then transferred into naïve mice that were rechallenged either immediately or 30 days after transfer with LM infection. The distal daughter cells (CD8^lo^) exhibited superior protection against delayed secondary infection. Overall, the study demonstrated that the asymmetric division might have an important role in T-cell biology and set the selection of experimental techniques that are now widely used in the field.

## Conserved asymmetric division mechanisms in T cells

Asymmetric division is an established field within developmental biology and multiple mechanistic details are already elucidated (1, 16, 17). In this section, we discuss studies demonstrating that asymmetric division mechanisms are shared between T cells and other cell types.

Oliaro et al. (2010) asked whether the asymmetric division mechanisms present in other cell types were conserved and also played a role in T cells (19). For that, they cocultured CD8+ T cells and peptide-pulsed C57BL/6 BM-derived Dendritic cells (DCs) with or without IL-2, IL-15, or aPKC inhibitor. Time-lapse microscopy was performed every 2 min at 40 h of T-cell coculture with DCs to observe T-cell–DC interactions. To detect the polarization of molecules during early and late mitosis, T-cell–DC conjugates were fixed, stained, and visualized by C.M. The polarity machinery (aPKC, Par-3, Scribble, DlgF, Pins, and Prox1), PKCθ, Numb, CD8, LFA-1, CD45, and tubulin were stained for confocal microscopy.

The study found that T cells divide while attached to APCs, with the distal daughter inheriting aPKC, Par-3, Numb (a Notch inhibitor), and Pins, whereas the proximal daughter inherited Scribble, DlgF, and PKCθ. Interestingly, in this model, synaptic proteins (CD8 and LFA-1) were not polarized at early or late mitosis, which contrasts the other asymmetric division studies. The study also concludes that memory of contact with APC is not sufficient for polarity establishment during division and that T cells must divide on DCs to do so.

Next, Metz et al. (2015) looked at the role of atypical PKCs (PKCζ and PKCι) in T-cell asymmetric division (20). Naïve transgenic OT-I CD8+ T cells (WT and PKCζ- or PKCι-deficient) were labeled with CFSE and transferred into C57BL/6 mice 24 h after LM–ovalbumin (OVA) infection. Thirty-six hours after transfer, undivided CD8+ T cells were sorted and analyzed by confocal microscopy with CD8, IFNγR, IL-2Rα, T-bet, proteasome, LFA-1, and tubulin staining. In addition, first-division daughters were either subject to single-cell PCR analysis of 96 genes or sorted into IL-2Rα^hi^ and IL-2Rα^lo^ and transferred into infection-matched mice rechallenged with a secondary infection 50 days after transfer.

The study showed that the loss of either aPKC isoform did not affect synapse formation but led to the disruption of proteasome, T-bet, IL-2Rα, and IFNgR asymmetry during first division. First-division cells without either PKCζ or PKCι lost their transcriptional heterogeneity and increased expression of IL-2Rα and Interferon regulatory factor 4 (IRF4). In line with that, T cells lacking either PKCζ or PKCι formed fewer memory precursor cells and failed to proliferate upon secondary infection rechallenge yet still were able to clear the infection.

The follow-up study by the same group looked at the effect of complete loss of atypical PKCs (PKCζ and PKCι) on asymmetric division (21). Naïve transgenic OT-I CD8+ T cells (WT and PKCζ-PKCι–deficient) were labeled with CFSE and transferred into C57BL/6 mice 24 h after LM-OVA infection. Thirty-six hours after transfer, undivided CD8+ T cells were sorted and analyzed by confocal microscopy IL-2Rα, IFNγR, T-bet, IRF4, proteasome, and tubulin staining as well as analyzed by flow cytometry (Commonly used BrdU – Bromodeoxyuridine (BrdU) and caspase-3/7; mitochondrial membrane potential: Δψm). First-division daughters were sorted into IL-2Rα^hi^ and IL-2Rα^lo^ and transferred into infection-matched mice rechallenged with a secondary infection 50 days after transfer.

This study revealed that the complete loss of aPKC (PKCζ-PKCι–deficient) abrogated asymmetric division and resulted in a 20% reduction in CD8+ T-cell number during primary antitumor response. On days 5 and 7 after infection, aPKC-deficient T cells had the same levels of T-bet GZMB, Tumor necrosis factor-alpha (TNFa), and IFNγ as wild-type (WT) cells but lower levels of Eomes, Bcl2, and IL-2. Moreover, on day 50 after infection (aPKC-deficient), the number of CD8+ T cells displayed a 50%–70% reduction compared with that of WT cells. Importantly, IL-2Rα^lo^ T cells (either aPKC-deficient or WT) expanded better during secondary response. That means that aPKС-driven asymmetry is dispensable once cells have acquired differential amounts of IL-2Rα.

Shifting the focus, Emurla et al. (2021) looked at the role of ER-membrane lateral diffusion barrier in asymmetric division regulation in T cells (22). During mitosis, the ER membrane is divided into two distinct domains, each allocated to the respective future daughter cell (12–14). This segregation is achieved by restricting lateral diffusion through a barrier present in the future plane of cleavage. The distinct domains formed in the ER membrane promote unequal segregation of various proteins.

To address this question, the authors transduced naïve CD8+ T cells from B6 mice with Sec61α-green fluorescent protein (GFP) (ER membrane reporter) and KDEL-modified GFP (ER luminal reporter) to track the exchange of the ER related proteins between daughter T cells during mitosis. The cells were activated with anti-CD3/CD28/Intercellular Adhesion Molecule 1 (ICAM1) for 24 h, and fluorescence loss in photo-bleaching (FLIP) live confocal microscopy (CD8 and mitochondria staining) was used to monitor these ER membrane and lumen reporters in dividing cells.

The authors discovered that, during TCR-driven (but not homeostatic) proliferation, a fraction of dividing CD8 T cells created a lateral diffusion barrier in the ER membrane at the future division site. This ER diffusion barrier correlated with CD8 and mitochondria asymmetry and required PKCζ signaling for establishment.

To summarize, these studies demonstrated that T cells indeed share asymmetric division mechanisms such as aPKC enrolment and ER-membrane lateral diffusion barrier with other cell types.

## Proximal TCR signaling role in asymmetric division

Expectedly, the strength and duration of TCR signaling have an impact on asymmetric division incidence. In this section, we discuss studies that connect the asymmetric division to TCR signaling.

King et al. (2012) were the first to investigate the role of TCR affinity in the asymmetric division of CD8+ T cells (23). They transferred CFSE-labeled naïve transgenic OT-1 CD8+ T cells into C57BL/6 mice subsequently immunized with peptide and Lipopolysaccharides (LPS). Twenty-four to 36 h after transfer. The cells were either directly analyzed or sorted into proximal (CD8^hi^) and distal (CD8^lo^) daughters followed by coculture with peptide-pulsed DCs for 20 min. Alternatively, naïve CD8+ T were cocultured with peptide-pulsed DCs. After 4 h or 24 h, T-cell–APC conjugates were mechanically disrupted, and T cells were further cultured with LPS-activated DCs in the absence of peptide for an additional 44 h or 24 h, respectively. CD8, LFA-1, Scribble, aPKC, Numb, and tubulin were stained for confocal microscopy analysis. The authors found that high-affinity antigens resulted in long T-cell–APC contacts, leading to asymmetric division, whereas low-affinity antigens or short-term (4 h) contact with APC led to impaired polarity and asymmetric division. Distal daughters had a higher proportion of memory precursor effector cells (MPECs) and Killer cell lectin-like receptor subfamily G member 1 (KLRG1) and IL-7Ra double-positive cells but lower SLECs. Proximal daughters formed more conjugates with DC during coculture and inherited more CD8 and LFA-1.

Gräbnitz et al. (2023) also focused on the role of TCR affinity in CD8+ T-cell asymmetric division (24). In this study, naïve transgenic P14 T cell factor 1 (TCF1)-GFP CD8+ T cells were activated by high- or low-affinity peptide-loaded DCs and, after 24 h, single-cell–sorted into 384-well plate. Then, time-lapse imaging (60-min interval) was performed for additional 24 h to record the first cell division, and, after 3 days of culturing, formed colonies were imaged and analyzed for fate acquisition. This work revealed that the strong TCR stimulation resulted in increased rates of asymmetric division, and single cells that underwent asymmetric division formed colonies consisting of both effector (CD62L−TCF1−) and memory precursor cells (CD62L+TCF1+). In contrast, upon weak TCR stimulation, asymmetric cell division did not lead to distinct cell fates, with activated cells exclusively forming colonies of either memory or effector precursor cells. Moreover, upon strong TCR stimulation, colonies from asymmetrically divided cells were enriched for TCF1-GFP+ memory precursor cells, whereas colonies derived from a symmetrically divided cells comprised few or no memory precursor cells. Using a separate *in vitro* system where P14 TCF1-GFP cells were activated by anti-CD3/CD28/ICAM-1 for 36 h to 40 h and then cultured with IL-2, IL-7, and IL-15, the authors showed that TCF1 downregulation started after two to three cell divisions, whereas CD62L downregulation required at least four divisions.

Along the lines of Gräbnitz et al. (2023), another study addressed division speed during T-cell activation phase and correlated this speed to T-cell differentiation trajectories (25). The authors activated naïve CD8+ T cells from C57BL/6 mice with plate-bound anti-CD3/CD28 antibodies and IL-2 either continuously or for 24 h. Subsequently, cells were monitored via live-cell imaging for 5 days (around 10 divisions) with or without IL-2Rα and CD62L staining. The authors found that, during the initial two to three divisions, T-cell clones divided homogenously fast but later segregated into fast- or slow-cycling subsets. Approximately 40% of T-cell clones gave rise to both fast and slow subsets with the difference in IL-2Rα expression (and IL-2 signaling) driving that asymmetry.

As LFA-1–mediated adhesion is required for the optimal synapse formation and TCR signaling, another study looked into causes and effects of LFA-1 asymmetric distribution in CD8+ T cells (26, 27). Naïve CD11a-GFP (α subunit of LFA-1) CD8+ T cells were either transferred into recipient mice infected with influenza-OVA 24 h later or activated by peptide-pulsed DCs on ICAM1-coated plates for 30 h and analyzed by live imaging and flow cytometry. First-division CD8+ T cells from infected mice were sorted into LFA-1^hi^ and LFA-1^lo^ populations 56 h after infection and transferred into new hosts inoculated with influenza-OVA. This work revealed that, upon antigen encounter, intracellular LFA-1 translocated to the immune synapse in T cells, and this LFA-1 redistribution was maintained during the division, resulting in an unequal LFA-1 inheritance. LFA-1^hi^ daughter cells formed stable and prolonged interactions with APCs, whereas LFA-1^lo^ exhibited increased migration capacity. Consistent with the prior reports, LFA-1^hi^ T cells showed higher expression of effector genes and LFA-1 asymmetry largely correlated with CD8 segregation. *In vivo*, LFA-1^hi^ and LFA-1^lo^ daughter cells equally expanded in the course of the primary infection, yet LFA-1^hi^ daughters showed impaired memory formation.

Together, these studies show that the strong and prolonged TCR engagement is required for the asymmetric division and influences the speed of subsequent cell division.

## Asymmetric division in differentiated T-cell subsets

Asymmetric division is not an exclusive feature of naïve T cells as other T cells subsets can also divide asymmetrically. In this section, we discuss studies that looked at the asymmetric division in differentiated T cells including memory and effector subsets.

Ciocca et al. (2012) were the first to address this question and looked at the asymmetric division in the memory subset of CD8+ T cells (28). They harvested transgenic memory P14 CD8+ T cells from mice 60 days after acute lymphocytic choriomeningitis (LCMV) infection and labeled with CFSE before transferring into C57BL/6 mice that were then infected with LM-gp33. Forty-two hours to 46 h after LM-gp33 rechallenge, memory CD8+ T cells were sorted and analyzed by confocal microscopy [CD3, IFNγR, IL-2Rα (CD25), T-bet, Eomes, and CD62L] and flow cytometry (CFSE, IL-2Rα, CD62L, and T-bet). The authors found that CD3, IFNγR, IL-2Rα, and T-bet segregated to the same side of the dividing cell, whereas EOMES did not show asymmetric segregation. About 50% of cells showed asymmetry in the above markers, whereas only 12% showed transcription factor EOMES asymmetry. Asymmetry of IL-2Rα, CD62L, and T-bet was preserved among cells that divided up to three times, after which all cells had higher levels of IL-2Rα and T-bet and lower levels of CD62L. Furthermore, in premitotic and mitotic memory T cells, PKCζ polarized to the side of the synapse, which was contrary to what was observed in naïve cells. The researchers also found that Central memory T cells (T_cm_) (CD62L^hi^) were more prone to asymmetry than Effector memory T cells (T_em_) (CD62L^lo^).

Next, Borsa et al. (2019) examined the asymmetric division at different differentiation states in human and murine CD8+ T cells (29). They transferred naïve transgenic P14 CD8+ T cells into mice followed by acute LCMV or chronic LCMV infection. Naïve, SLEC (KLRG1+ CD127−), MPEC (KLRG1− CD127+), effector memory (CD44+ CD62L− 30 days after acute LCMV), central memory (CD44+ CD62L+), PD1^int^, and PD1^hi^ (30 days of chronic LCMV) murine T cells or human naïve (CD45RA+ CD62L+) and central memory (CD45RO+ CD62L+) CD8+ T cells were activated by plate-bound anti-CD3/CD28/ICAM1 (with or without rapamycin, Akt PKC, and Hypoxia-inducible factor 1-alpha (HIF1a) inhibitors or Sphingosine-1-phosphate (S1P) agonist). Thirty hours to 36 h after activation, conjoined daughters were analyzed by confocal microscopy with CD8, T-bet, and tubulin staining. The study revealed that asymmetric division correlated with cellular stemness level. Naïve, memory, and progenitor-exhausted T cells were able to divide asymmetrically, whereas SLECs or terminally exhausted T cells were not. Moreover, mammalian target of rapamycin (mTOR) (12 h after activation) or HIF1a inhibition enhanced division asymmetry, whereas S1P receptor agonist did otherwise. Interestingly, mammalian target of rapamycin (mTOR) inhibition known to promote memory T-cell differentiation did so only if T cells divided asymmetrically. This study was also the first to show that human naïve (CD45RA+ CD62L+) and central memory (CD45RO+ CD62L+) CD8+ T cells can divide asymmetrically *in vitro*.

In a follow-up study, the same group investigated ageing impact on asymmetric division in T cells (30). CD8+ T cells (bulk, naïve CD44^lo^, or T virtual memory CD44^hi^ CD49d^lo^) isolated from naïve young, middle-aged, and old P14 mice were activated (with or without mTOR inhibition) for 36 h by plate-bound anti-CD3/CD28/ICAM1 and analyzed by confocal microscopy with CD8, T-bet, tubulin, and blue-fluorescent DNA stain (DAPI) staining. CD8 polarization was used as an asymmetric division readout. The authors demonstrated that virtual memory (CD44^hi^ CD49d^lo^) CD8+ T cells from both young and aged mice retained the ability to divide asymmetrically, whereas naïve CD8+ T cells from old mice largely lost this ability. Interestingly, transient Akt or mTOR inhibition had no impact on asymmetric division rates in virtual memory T cells yet increased asymmetric division rates in naïve T cells from both young and old mice.

Another related study investigated asymmetric segregation of lytic machinery during T cells division (31). Here, human bulk, memory, or clonally expanded CD8+ T cells were activated with immobilized anti-CD3/CD28/ICAM1 for 72 h and analyzed by imaging flow cytometry with CellTrace Violet (CTV; to trace divisions and total protein content), nuclear stain SYTOX orange (to identify bi-nucleated cells in anaphase and telophase), CD107a, perforin, GZMB, T-bet, c-Myc, and tubulin staining. CD107a asymmetry was confirmed by 3D confocal laser scanning microscopy. The authors discovered that the lytic granules in human CD8+ T cells distribute asymmetrically at each division (in 20% of telophasic cells), yet this asymmetry was not related to the traditional asymmetric division mechanics. Such an unequal lytic granule segregation contributed to the heterogeneity in the cytotoxic capacity of clonally expanded T cells. Contrary to other asymmetric cell division (ACD) reports, the authors did not observe asymmetric distribution of T-bet and c-Myc. This can potentially be explained by the longer activation period (72 h vs. traditional 36 h) during which T cells could have completed several rounds of division.

To summarize, although asymmetric division clearly correlates with T-cell stemness (early differentiation stages), naïve T cells lose this ability with age.

## Asymmetric distribution of fate-determining factors in T cells

The fundamental question in the field is whether the asymmetric division regulates differentiation and fate decision in T cells. As T-cell differentiation trajectories are driven by the distinct fate-determining proteins and transcription factors, here, we discuss several studies elucidating their asymmetric inheritance.

To begin with, Chang et al. (2011) focused on asymmetric segregation of transcription factor T-bet and proteasome in CD8+ T cells (32). They transferred naïve transgenic P14 CD8+ T cells labeled with CFSE into C57BL/6 mice 24 h after gp33-41 Listeria infection. Thirty-six hours to 48 h after transfer, undivided CD8+ T cells or first-division daughter cells were harvested and analyzed by confocal microscopy and flow cytometry. In addition, naïve CD4+ T cells from C57BL/6 mice were activated by plate-bound anti-CD3/CD28/ICAM1-Fc proteins and analyzed 28 h to 32 h after activation. T-bet, CD3 aPKC, IFNγR, and tubulin were stained for confocal microscopy analysis. The authors demonstrated that aPKC was a driving force in asymmetric proteasome segregation, directing the proteasomes to the distal daughter cell. T-bet asymmetry was detected in 66% of cells, and the phosphorylation at Y525 by ITK triggered its proteasomal degradation in the distal daughter cell during mitosis.

Widjaja et al. (2017) continued to elucidate proteasome role in CD8+ T-cell fate acquisition (33). In this study, naïve transgenic OT-1 CD8+ T cells labeled with CFSE were transferred into C57BL/6 mice 24 h after LM-OVA (for some experiments, cells were pretreated for 4 h with proteasome activator or inhibitor prior to transfer). Forty-five hours after transfer, proteasome activity in first-division cells (second brightest CFSE) was analyzed by flow cytometry with distal daughters identified by CD62L^hi^ IL-2Rα^lo^ proteasome^hi^ phenotype. For memory formation experiments, these CD8+ T cells were analyzed 50 days after infection and rechallenged with LM-Ova. *In vitro*, naïve CD8+ T were activated by anti-CD3/CD28 antibodies in the presence of proteasome activator or inhibitor. This work revealed that pharmacological proteasome activation in first-division daughters led to reduced c-Myc expression. Brief proteasome inhibition prior to adoptive transfer impaired T memory formation *in vivo*, whereas proteasome activation in the same setting promoted T-cell memory formation.

With its role in T-cell differentiation, PI3K/mTOR signaling axis has been extensively studied in the context of the asymmetric division. Pollizzi et al. (2016) focused on mTORC1 signaling and activated naïve CD8+ T cells either by plate-bound anti-CD3/CD28/ICAM1 or by peptide-pulsed Bone marrow (BM)-derived DCs (34). Then, CD8^hi^ or CD8^lo^ T cells were transferred into mice infected with vaccinia-OVA virus same day or 21 days after transfer. For *in vivo* activation, naïve transgenic OT-1 CD8+ T cells were labeled with eFluor 450 and transferred into C57BL/6 mice followed by LM-OVA infection 24 h after transfer. Forty-eight hours after infection, first-division daughters (second brightest eFluor peak) were sorted and analyzed by confocal microscopy (p-S6, c-Myc–GFP, MitoTracker, RagC, LAMP-2, and CD98) and flow cytometry [CD44, CD69, IL-2Rα, T-bet, CD62L, (p)-TSK2, p-S6, p-4E-BP1, and c-Myc]. The authors discovered that 60% of first daughters showed asymmetry in activated mTORC1 (mTOR targets p-S6 and p-4E-BP1 as readout) and that increased partitioning of CD98 (which increased amino acid flux an RagC-mediated mTOR recruitment to lysosomes) to CD8^hi^ daughter likely caused mTOR asymmetry. CD8^lo^ daughter was less glycolytic but preserved more mitochondria (downregulated c-Myc, GLUT1, HK2, upregulated CPT1a, VDAC, NDUFB8, SDHA mitochondria mass, and DNA) and more antiapoptotic molecules (BCL2 and BCL-xL). Function-wise, unlike CD8^hi^, CD8^lo^ significantly expanded after secondary infection rechallenge showing that CD8^lo^ daughters preferentially from memory T cells. Importantly, asymmetric division of CD8 marker was not controlled by mTORC1 activity and CD69 and CD44 did not show asymmetric distribution.

At the same time, Verbist et al. (2016) looked into mTORC1 and c-Myc asymmetric distribution in CD8+ T cells (35). In this study, naïve c-Myc–GFP CD8+ T cells labeled by CTV were activated by plate-bound anti-CD3/CD28/ICAM1 and 35 h later were pulsed with BrdU for 1 h. Thirty-six hours after activation c-Myc asymmetry was assessed by confocal microscopy (mitotic and conjoined daughters) and flow cytometry. Naïve transgenic OT-1 CD8+ T cells were cultured with peptide-pulsed BM-derived DCs, and time-lapse microscopy of T cells dividing on DCs was performed. For *in vivo* T-cell memory assessment, c-Myc^hi^ or c-Myc^lo^ OT-I first daughters were transferred into separate hosts (naïve or infected with HKx31-OVA influenza) that were rechallenged with a heterosubtypic virus 2 weeks later.

The study concluded that proximal daughters expressed more c-Myc, Numb, Scribble, IL-2Rα, CD98, p-S6 p-70S6K, and p-FOXO1 and were more glycolytic, whereas distal daughters expressed more PKCζ. Moreover, asymmetric CD98 and mTORC1 activity sustained the asymmetric distribution of c-Myc. c-Myc was not polarized before and during mitosis obtaining asymmetry only after formation of daughter cells. In subsequent divisions, asymmetry dissipitated. Contrary to the previous studies (32, 34), proteasome inhibition did not affect asymmetry and no differences in mitochondrial mass or DNA were detected in first daughters. Function-wise, in line with the previous study, c-Myc^lo^ daughters proliferated less after first division but expanded significantly better after secondary infection rechallenge.

Liedmann et al. (2022) continued to investigate upstream and downstream regulators of c-Myc asymmetry in CD8+ T cells (36). In this study, stochastic optical reconstruction microscopy (STORM) was applied either to OT-I CD8+ T cells stimulated with peptide-pulsed APCs or to lymph node tissue 24 h after immunization. The authors discovered that, through the physical association with the active mTORC1, Eukaryotic initiation factor 4F (eIF4F) complex polarized toward MTOC and was preferentially accumulated in the proximal daughter. As eIF4F complex promotes c-Myc translation, the proximal daughter expressed higher levels of c-Myc protein than the distal daughter.

In a separate experiment by the same authors, CTV-labeled GFP–c-Myc and OT-I CD8+ T cells were activated by peptide-pulsed APCs for 36 h and first-division c-Myc^hi^ and c-Myc^lo^ cells (highest and lowest 20% GFP–c-Myc) were sorted and transferred into congenially distinct animals infected with OVA-expressing influenza A virus. Thirty days later, animals were rechallenged with another strain of OVA-expressing influenza A virus, and splenic CD8+ T cells were analyzed by flow cytometry. The authors discovered that the sorted c-Myc^hi^ CD8+ T cells failed to differentiate into memory cells and did not respond to the secondary infection. To assess Asymmetric division (AD) contribution to transcriptional diversity among first-division T cells, the authors employed an endogenous barcode transgenic mouse model. CTV-labeled OT-I BCM CD8+ T cells were activated by peptide-pulsed APCs for 36 h, and first-division OT-I BCM CD8+ T cells expressing a barcode (GFP-bcm+) were sorted and sequenced. On the other hand, for *in vivo* stimulation, CTV-labeled OT-I CD8+ T cells were transferred into recipient mice immunized with OVA peptide. Twenty-four hours later, barcode-containing cells were isolated from spleens and lymph nodes, sorted, and sequenced. This effort revealed that first-division daughter cells often displayed transcriptional variation, and mTORC1 and c-Myc signaling levels correlated with this transcriptional variation.

Another recent study focused on c-Myc–Canonical BAF complex (cBAF) interplay during T-cell asymmetric division (37). Here, CTV-labeled c-Myc-GFP and OT-I CD8+ T cells were activated by anti-CD3/CD28/ICAM1 for 28 h, fixed, and analyzed by confocal microscopy with cBAF (SMARCB1, ARID1A, and BRG1), tubulin, laminin, and Hoechst staining. Alternatively, first-division c-Myc^hi^ and c-Myc^lo^ (or CD98^hi^ and CD98^lo^) T cells were sorted for immunoblot analysis and Assay for Transposase-Accessible Chromatin using sequencing (ATAC-seq). The authors discovered preferential cBAF distribution into c-Myc^hi^ CD98^hi^ daughter cells that also had increased chromatin accessibility in the promoter, intronic and intergenic regions. Heterozygous loss of c-Myc reduced the asymmetric distribution of chromatin accessibility, which pointed out to c-Myc central role in this process. On the other hand, no asymmetry in PBAF complex was observed.

Chen et al. (2018) addressed early asymmetry events up to metaphase of T-cell mitosis along with PI3K role in asymmetric division (38). Naïve P14 CD8+ T cells were activated with gp33 peptide-pulsed splenocytes. Three days later, T cells completed several rounds of division and were analyzed by confocal microscopy with CD3, IRF4, PIP3, TCF1, Glut1 tubulin, and DNA staining. For *in vivo* analysis, naïve TCF1-GFP P14 CD8+ T cells were transferred into naïve mice followed by gp33 Listeria infection. Four days to 5 days after infection, TCF1-GFP^hi^ cells were sorted and analyzed by confocal microscopy. The authors discovered that CD3 (up to 90%), PIP3 (60%–80%), and Glut1 (60%) segregated to MTOC pole in metaphase of T-cell division. Moreover, PI3K inhibition resulted in defective silencing of TCF1, reduced cell surface trafficking of Glut1, and defective CD3 polarity during metaphase. The authors concluded that PI3K signaling plays an essential role in asymmetric distribution of at least some proteins (CD3 and Glut1) in T cells.

Although the asymmetric division is usually studied in the first-division daughter cells, several studies addressed this phenomenon at later divisions. As discussed below, some proteins and transcription factors show asymmetric segregation not during the first-division but at later time points.

Lin et al. (2015) addressed asymmetry at later divisions as well as asymmetry in fate decision transcription factors IRF4, c-Myc, TCF1, and forkhead box protein O1 (FOXO1) (39). The authors transferred naïve transgenic P14 CD8+ T cells into mice followed by gp33 Listeria or acute LCMV infection. Alternatively, naïve P14 CD8+ T cells were activated with gp33 peptide-pulsed splenocytes 3 days to 4 days after transfer or *in vitro* activation conjoined daughters (not necessarily first-division) were analyzed by confocal microscopy with IRF4, FOXO1, TCF1, c-Myc, tubulin, and DNA staining. The study found that asymmetry in IRF4 and c-Myc was observed within the first three cell divisions. Conjoined daughters within the first three cell divisions generally exhibited equal TCF1 expression and FOXO1 nuclear localization, but, at later time points, asymmetry in TCF1 (55% *in vitro* activation, 62% - Listeria, 75% - LCMV), and FOXO1 was observed. In line with the fate decision paradigm, TCF1 and nuclear FOXO1 segregated oppositely from IRF4 and c-Myc.

Nish et al. (2016) investigated the asymmetry in memory-associated transcription factor TCF1 in CD4+ T cells (40). TCF1-GFP or WT and naïve OT-II CD4+ T cells labeled with a cell proliferation dye were transferred into recipient mice, which were subsequently infected with PR8-OVA influenza virus. Four days later, OT-II+ CD4+ T cells were sorted and examined by confocal microscopy with TCF1, tubulin, and DNA staining. Alternatively, TCF1-GFP^hi^ and TCF1-GFP^low^ CD4+ T cells were sorted and restimulated with anti-CD3/CD28 *in vitro*. The authors found that TCF1^hi^ CD4+ T cells gave rise to both TCF1^hi^ and TCF1^lo^ progeny, whereas TCF1^lo^ cells only generated TCF1^lo^ progeny. Although TCF1 distribution was symmetric during metaphase, 74% (56% for *in vitro* differentiation) of conjoined daughters at telophase showed TCF1 asymmetric distribution. Similar to CD8+ T cells (39), CD4+ T cells began to silence TCF1 after four to five divisions. In line with the previous studies in CD8+ T cells (38, 39), the authors concluded that asymmetric PI3K signaling during telophase drives TCF1 asymmetry in CD4+ T cells.

Altogether, these studies suggest that the asymmetric division indeed plays a role in T-cell differentiation through the asymmetric segregation of fate-determining proteins and transcription factors.

## Transcriptional and epigenetic characterization of T-cell asymmetric division

As only a limited number of features can be analyzed by the methods routinely used in the asymmetric division field (such as confocal microscopy or flow cytometry), complementary sequencing methods quickly enter the field and we discuss them in this section.

Arsenio et al. (2014) were the first to look at the transcriptional heterogeneity among asymmetrically divided T cells (41). They transferred naïve transgenic OT-1 CD8+ T cells labeled with CFSE into C57BL/6 mice followed by LM-OVA 24 h later. Thirty-six hours after transfer, undivided CD8+ T cells were sorted, fixed, and analyzed by confocal microscopy with IL-2Rα, CD62L, and tubulin staining. First daughters (second brightest CFSE peak) were sorted into CD62L^lo^ IL-2Rα^hi^ or CD62L^hi^ IL-2Rα^lo^ and transferred into naïve mice infected with LM-OVA 48 h before. Fifty days after primary infection, these mice were rechallenged with LM-OVA. In addition, first-division daughters, day 3, day 5, day 7 (SLEC and MPEC separately), and day 45 (T_cm_ CD44^hi^CD62L^hi^ and T_em_ CD44^hi^CD62L^lo^) cells were sorted and subject to single-cell PCR analysis of 96 genes. The authors observed significant heterogeneity among cells isolated early after infection (first-division and day 3 after infection) compared with later time points, and IL-2Rα and CD62L distributed asymmetrically in 60% of conjoined daughters. On the functional level, CD62L^hi^ IL-2Rα^lo^ daughter cells generated four-fold more central memory T cells and expanded 10-fold more at secondary infection rechallenge.

Next, the same group took advantage of Single cell RNA and Chromatin immunoprecipitation (scRNA and CHIP) sequencing to elucidate transcriptional and epigenetic regulation during asymmetric division (42). Similarly, naïve transgenic P14 CD8+ T cells (WT or Ezh2-deficient) were labeled with CFSE and transferred into C57BL/6 mice 24 h after gp33-41 Armstrong LCMV infection. First-division daughters, day 4, day 7, and day 42 T cells were sorted and subject to scRNA sequencing. Moreover, H3K27me3 ChIP-seq and EZH2 ChIP-seq on day 8 (SLEC) and day 60 T cells were performed. On the basis of transcriptomic data, two distinct populations were observed in first-division daughter cells. One population was transcriptionally closer to naïve and central memory T cells, whereas the other was closer to day 4 and day 7 effector T cells. In line with this, Ezh2 protein expression was greater in CD8^hi^ first-division daughters in which it repressively methylated (H3K27me3) genes associated with T-cell memory. The authors concluded that the T-cell fate might already be transcriptionally and epigenetically imprinted after the first-division. In addition, the authors also identified 89 new potential regulators of CD8+ T-cell fate decision.

Quezada et al. (2023) also investigated transcriptional and epigenetic divergence of first-division CD8+ T cells during acute and chronic LCMV infection (43). Naïve transgenic P14 CD8+ T cells (WT or Ezh2-deficient) were labeled with CFSE and transferred into congenic mice infected with either Armstrong or C13 LCMV infection. Single-cell RNA and ATAC-seq was performed on day 2 (first-division), day 3, day 5, day 6, day 7, day 8, day 22, day 34, and day 60 after infection. This work revealed that, unlike first-division Arm CD8+ T cells (forming two distinct clusters: effector-like and memory-like), first-division C13 CD8+ T cells formed a single scRNA-seq cluster and expressed higher levels of NFAT (*NFATC1* and *NFATC2)*, PRC2 complex (*EZH2* and *SUZ12)*, and inhibitory receptors (*HAVCR2, LAG3*, and *PDCD1*). Intriguingly, on the epigenetic level, opposite trend was observed where first-division C13 CD8+ T cells formed three ATAC clusters as opposed to the single cluster in first-division Arm CD8+ T cells. The authors concluded that T-cell differentiation divergence between acute and chronic states is already imprinted at first division.

Together, these studies demonstrated the transcriptional and epigenetic heterogeneity as well as the existence of effector-like and memory-like clusters among first-division daughter cells.

## Geometric aspects of T-cell asymmetric division

**Figure 1:**
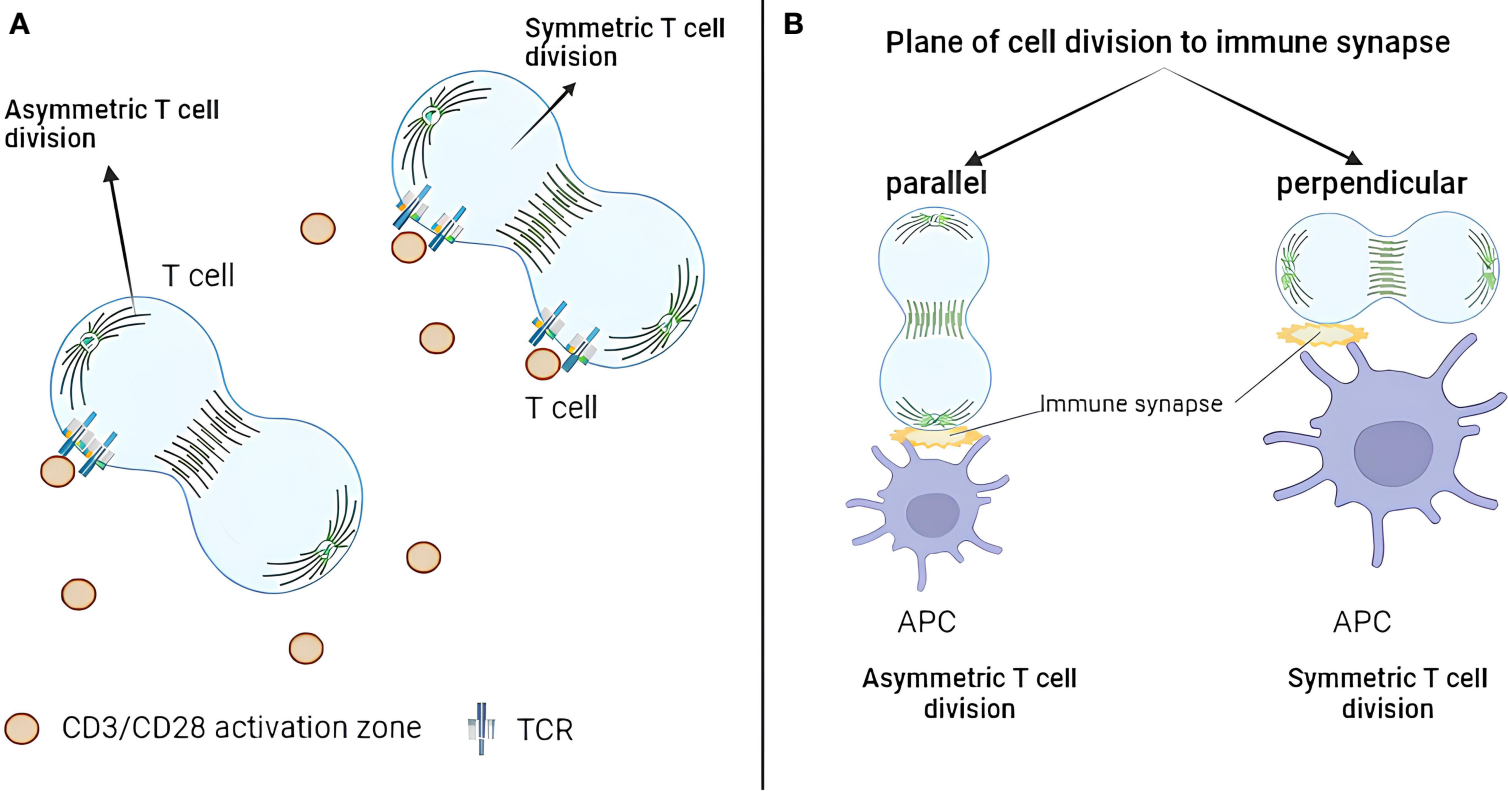
Geometric aspects of T-cell asymmetric division. **(A)** TCR exhibits asymmetric distribution only when one daughter cell remains in contact with a TCR activation site. Conversely, when both daughter cells contact activation sites during division, TCR distributes symmetrically. **(B)** Only 10% of dividing CD4+ T cells demonstrate division plane parallel to the immune synapse. The asymmetric division happens when only one synapse is present, which is parallel to the division plane. TCR, T-cell receptor.

Although the geometry of asymmetric division has not been explored extensively, two studies address this question. Jung et al. (2014) investigated the role of additional immune synapses during T-cell asymmetric division (44). They purified naïve murine CD4+ T cells from C57BL/6 mice and loaded them on immunological synapse arrays (ISAs). ISA is an ICAM-1–coated surface on which T-cell activation sites (anti-CD3/CD28) are dispersed with equal distance (15 μm or 25 μm). After 32 h of incubation, T cells were fixed and analyzed by fluorescence microscopy with stained TCR, T-bet, PKCζ, tubulin, and DAPI staining. The difference between TCR-integrated fluorescence intensity of each daughter cell was used as a metric for asymmetric division. The work revealed that, only if one daughter cell remained in contact with a TCR activation site, TCR molecules will be asymmetrically distributed (**Figure 1A**). On the other hand, TCR molecules were distributed symmetrically if both daughter cells contacted activation sites during division. Asymmetric division incidence did not depend on the amount of TCR activating signals. The authors concluded that TCR signaling drives repolarization of key molecules required for T-cell asymmetric division. However, this conclusion should be taken with a grain of salt as asymmetric inheritance of TCR could be caused by TCR clustering at the site of activation and not because of the true asymmetric division mechanics.

Alampi et al. (2022) looked at the division plane orientation in CD4+ T cells in the Type 1 diabetes (T1D) context (45). Fluorescence-activated cell sorting (FACS)-sorted naïve CD4+ T cells from patients with T1D or healthy controls were labeled with CFSE, stimulated with glutamate decarboxylase 65 (GAD65) protein loaded autologous DCs for 48 h (with or without IL-7) and analyzed by confocal or time-lapse microscopy with CD4 and Hoechst staining. First-division CD45RA^hi^ and CD45RA^lo^ T cells were sorted and cultured for 24 h before the analysis by confocal microscopy with CD184, CD95, CD127, CD132, HLA-DR, CD3, CD4, CD62L, GLUT1, β-catenin, and the mitochondrial mass staining. The authors concluded that around 10% of dividing CD4+ T cells had division plane parallel to the immune synapse, and proximal first-division daughters showed increased GLUT1 and CD184 but reduced CD45RA, β-catenin, and mitochondrial mass (**Figure 1B**). In other words, only 10% of the cells produced proximal and distal daughters, whereas, in 90% of the cases, both daughters kept contact with the APC. This is of note as orientation of the division plane was largely neglected by the field. On the other hand, no asymmetry between CD45RA^hi^ and CD45RA^lo^ T cells in CD95, CD127, CD132, CD3, CD4, CD62L, and HLA-DR was observed. Moreover, IL-7 treatment upregulated GLUT1 expression and increased asymmetric division rates in autoreactive T cells from patients with T1D.

Together, these studies showed that the asymmetric division in T cells is favored when only one synapse is present and is parallel to the division plane. Although not yet explicitly demonstrated, these findings can likely be extended to CD8+ T cells. As an indirect evidence, ACD rates in CD8+ T cells are low and can potentially by explained by the low frequency of parallel synapse formation.

## Conclusions, unanswered questions, and future perspectives

**Figure 2:**
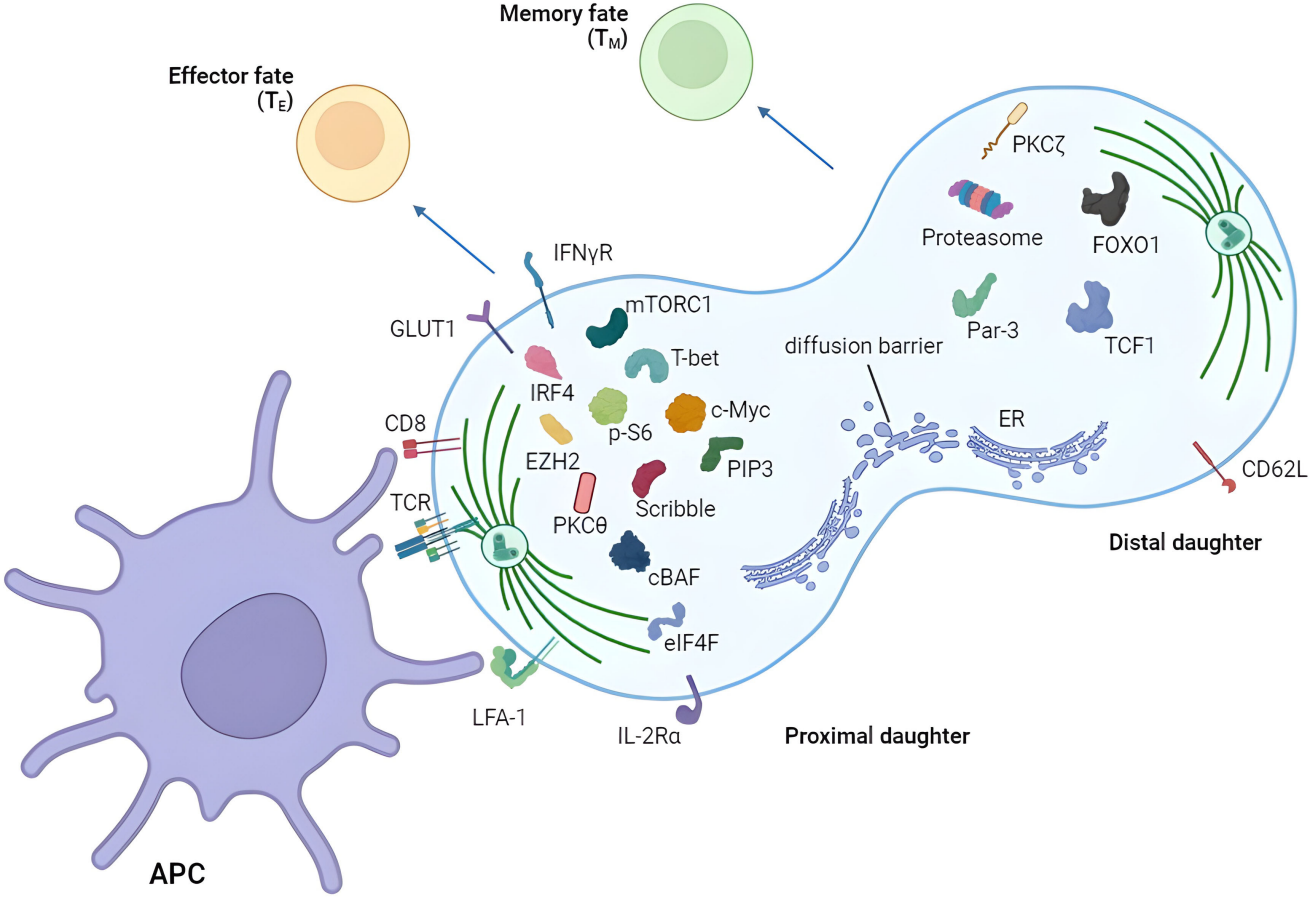
Asymmetric segregation of receptors and fate-determining factors in T cells. T cell can divide asymmetrically after the activation by APC showing unequal distribution of various proteins between proximal and distal daughters. Proximal and distal daughters are prone to become effector and memory T cells respectively. APC, antigen-presenting cell.

For reader’s convenience, we have summarized discoveries in the asymmetric division field (**Figure 2**; **Table 1**). However, several unanswered questions remain.

**Table 1:** T cell asymmetric division publications.

Publication	Readout to differentiate daughter cells	Experimental systems	Effector-like daughters	Memory-like daughters	Findings	Citation
Chang et al. (2007)	CD8 expression	LM-gp33 and Leishmania infection Confocal microscopy and flow cytometry	CD3, CD4/CD8, LFA-1, IFNγR, Scribble	PKCζ, Superior protection against secondary infection	1) Bimodal distribution of CD62L, IL-2Rα, GZMB, and LFA-1 in daughter cells. 2) No asymmetry without contact with APC.	(18)
Oliaro et al. (2010)	Microscopy	GFP-expressing OT-1 CD8+ T cells coculture with DCs Confocal and time-lapse microscopy	PKCθ, Scribble, and DlgF	aPKC, Par-3, Pins, and Numb	1) CD8, LFA-1 are NOT polarized during mitosis. 2) T cell must divide ON APC to establish division polarity.	(19)
Chang et al. (2011)	CD8 expression	LM-gp33, CD4+ T cells activated by immobilized anti-CD3/CD28/ICAM1 Confocal and time-lapsed microscopy	T-bet	Proteasome	1) aPKC drives asymmetric proteasome segregation. 2) p-Y525 T-bet degraded during mitosis by proteasome.	(32)
Ciocca et al. (2012)	Microscopy	Armstrong LCMV and LM-gp33, Confocal microscopy and flow cytometry	PKCζ (in memory cells), CD3, CD8, IFNγR, IL-2Rα, and T-bet	PKCζ (in naïve cells) and CD62L	1) EOMES does not asymmetrically segregate. 2) T_cm_ more prone to asymmetric division than T_em_. 3) Asymmetry of IL-2Rα, CD62L, and T-bet preserved up to three divisions.	(28)
King et al. (2012)	CD8 expression	OVA peptide immunization and OT-1 CD8+ T cells coculture with DCs Confocal microscopy and flow cytometry	CD8, LFA-1, Scribble, and Numb, SLEC (KLRG1+IL-7Ra–) phenotype and more conjugates with APC	PKC2, MPEC (KLRG1-IL-7Ra+) phenotype	1) High-affinity antigens led to long T-cell–APC contacts and asymmetric division. 2) Increased accumulation of proximal daughters during primary infection. 3) Short-term contact with APC not enough for polarity.	(23)
Jung et al. (2014)	TCR expression	CD4+ T cells activated by immunological synapse arrays (anti-CD3/CD28) with fluorescent microscopy	T-bet and TCR	PKCζ	1) Contact with activation site needed for TCR asymmetry. 2) Amount of activation signals has no impact on asymmetric division incidence.	(44)
Arsenio et al. (2014)	IL-2Rα CD62L expression	LM-OVA Confocal microscopy and sc-PCR (96 genes)	IL-2Rα	CD62L	1) Heterogeneity among first-division cells. 2) Better memory response by CD62L^hi^ IL-2Rα^lo^ daughter cells.	(41)
Lin et al. (2015)	Microscopy	Armstrong LCMV, LM-gp33, and CD8+ T cells activated by peptide-pulsed splenocytes Confocal microscopy	c-Myc and IRF4	FOXO1 and TCF1	1) Asymmetry in TCF1 and FOXO1 only after three divisions. 2) Equally high TCF1 and low c-Myc in rapamycin-treated conjoined daughters.	(39)
Metz et al. (2015)	IL-2Rα expression	LM-OVA Confocal microscopy and sc-PCR (96 genes)	IFNγR, IL-2Rα, and T-bet	Proteasome	Loss of either aPKC isoform: -does not affect synapse polarization; - disrupt proteasome, T-bet, IL-2Rα, and IFNgR asymmetry; - reduced heterogeneity and increased expression of IL-2Rα and IRF4 in first-division daughter cells.	(20)
Metz et al. (2016)	IL-2Rα expression	LM-OVA Confocal microscopy and flow cytometry	IL-2Rα, IFNγR, and T-bet	Proteasome	1) IL-2Rα^lo^ T cells (aPKC-deficient or WT) expanded better during secondary response. 2) aPKС-driven asymmetry is dispensable once cells acquired differential amounts of IL-2Rα.	(21)
Pollizzi et al. (2016)	CD8 expression	LM-OVA, vaccinia-OVA, and CD8+ T cells activated by immobilized anti-CD3/CD28/ICAM1 or peptide-pulsed DCs Confocal microscopy, flow cytometry, and immunoblotting	Activated mTOR (p-S6, p-4E-BP1), CD98, c-Myc, and LAMP-2	Mitochondria and BCL2	1) Asymmetric segregation of CD8 is not controlled by mTORC1 activity. 2) Better memory response by CD8^lo^ daughters. 3) No asymmetry in CD69 and CD44 expression.	(34)
Verbist et al. (2016)	c-Myc expression	Influenza-OVA and CD8+ T cells activated by immobilized anti-CD3/CD28/ICAM1 or peptide-pulsed DCs Confocal and time-lapse microscopy and flow cytometry	IL-2Rα, c-Myc, CD98, active mTORC1 (p-S6 p-70S6K), p-FOXO1) Numb, Scribble, and SLC1A5 More glycolytic and proliferative during primary response	PKCζ Improved proliferation during secondary response	1) c-Myc not polarized before and during mitosis and obtained asymmetry only after formation of daughter cells. In subsequent divisions, asymmetry dissipitated. 2) Asymmetric mTORC1 activity sustained the asymmetric assortment of c-Myc. 3) Proteasome inhibition did not affect asymmetry. 4) No differences in mitochondria in first-daughter cells.	(35)
Nish et al. (2016)	Microscopy	Influenza-OVA and CD4+ T cells restimulated by anti-CD3/CD28 Confocal microscopy	Th1 phenotype	TCF1	1) CD4+ T cells silenced TCF1 after four to five divisions. 2) Asymmetric PI3K signaling during telophase drove TCF1 asymmetry in CD4+ T cells.	(40)
Widjaja et al. (2017)	IL-2Rα CD62L expression	LM-OVA and CD8+ T cells activated by immobilized anti-CD3/CD28	IL-2Rα	CD62L and proteasome	1) Proteasome activation in first-division daughters led to reduced Myc expression and improved memory response. 2) Proteasome inhibition impaired memory response.	(33)
Capece et al. (2017)	LFA-1 expression and microscopy	Influenza-OVA and CD8+ T cells activated by peptide-pulsed DCs	CD8, LFA-1, and prolonged interactions with APCs	Increased migratory capacity	1) Intracellular LFA-1 translocated to immune synapse upon antigen encounter.	(26)
Kakaradov et al. (2017)	scRNA-seq	Armstrong LCMV scRNA-seq and CHIP-seq	EZH2		1) Memory-like and effector-like populations were observed after first-division. 2) Identified 89 new potential regulators of CD8+ T-cell fate decision.	(42)
Chen et al. (2018)	Microscopy	LM-gp33 and CD8+ T cells activated by peptide-pulsed splenocytes Confocal microscopy	CD3, PIP3, and GLUT1		1) PI3K inhibition resulted in defective silencing of TCF1, reduced Glut1 surface trafficking, and defective CD3 polarity during metaphase. 2) PI3K and GLUT1 asymmetry created metabolic bias between sibling cells.	(38)
Borsa et al. (2019)	Microscopy	Armstrong or C13 LCMV, and human or murine CD8+ T cells activated by immobilized anti-CD3/CD28/ICAM1 or peptide-pulsed DCs Confocal microscopy and RNA-seq	CD8 and T-bet	Improved proliferation during secondary response Increased migratory capacity to secondary lymphoid organs and white pulp in spleen.	1) Asymmetric division correlated with T-cell stemness. 2) mTOR or HIF1a inhibition enhanced asymmetry. 3) mTOR inhibition promoted memory formation only if T cells divided asymmetrically. 4) Rapamycin-enforced asymmetric division reinvigorated exhausted T cells. 5) Human naïve (CD45RA+ CD62L+) and T_CM_ (CD45RO+ CD62L+) divided asymmetrically.	(29)
Borsa et al. (2021)	CD8 expression	CD8+ T cells from young, middle-aged, and old mice activated by immobilized anti-CD3/CD28/ICAM1 Confocal microscopy	CD8, T-bet, and p-S6		1) Naïve CD8+ T cells from old mice lost ability to divide asymmetrically. 2) Virtual memory (CD44^hi^ CD49d^lo^) CD8+ T cells retained ability to divide asymmetrically.	(30)
Lafouresse et al. (2021)	Imaging flow cytometry and microscopy	Human CD8+ T cells activated by immobilized anti-CD3/CD28/ICAM1 Imaging flow cytometry and 3D confocal laser scanning microscopy	Not analyzed	Not analyzed	1) Lytic granules segregated asymmetrically at each division. 2) Lytic granules asymmetry contributed to heterogeneity in cytotoxic capacity. 3) No asymmetry in T-bet and c-Myc.	(31)
Emurla et al. (2021)	CD8 expression	CD8+ T cells activated by immobilized anti-CD3/CD28/ICAM1 FLIP live confocal microscopy	CD8 and mitochondria (both activity and total amount)		1) Lateral diffusion barrier in ER membrane at future division site. 2) ER diffusion barrier correlated with CD8 and mitochondria asymmetry and required PKCζ signaling for establishment	(22)
Liedmann et al. (2022)	c-Myc expression	OVA peptide immunization, Influenza-OVA, and CD8+ T cells activated by peptide-pulsed DCs Stochastic optical reconstruction microscopy (STORM), endogenous barcode transgenic mouse model (BCM), and scRNA-seq	MTOC, MTORC1, p-S6, polysomes, c-Myc, and eIF4F		1) c-Myc^hi^ CD8+ T cells failed to differentiate into memory cells. 2) mTORC1 and c-Myc signaling correlated with transcriptional variation in first-division daughter cells.	(36)
Guo et al. (2022)	CD98 expression	CD8+ T cells activated by immobilized anti-CD3/CD28/ICAM1 Confocal microscopy	cBAF and c-Myc		1) c-Myc^hi^ CD98^hi^ daughter cells had increased chromatin accessibility in promoter, intronic and intergenic regions. 2) No asymmetry in PBAF.	(37)
Alampi et al. (2022)	CD45RA expression	CD4+ T cells (healthy or patients with T1D) activated by peptide-pulsed DCs Confocal and time-lapse microscopy	GLUT1 and CD184	CD45RA, β-catenin, and mitochondria	1) Division plane parallel to IS only in 10% of CD4+ T cells. 2) No asymmetry in CD3, CD4, CD62L, CD95, CD127, CD132, and HLA-DR. 3) Through GLUT1 upregulation, IL-7 increased asymmetric division rates in autoreactive T cells from patients with T1D.	(45)
Plambeck et al. (2022)	Microscopy	CD8+ T cells activated by immobilized anti-CD3/CD28 Live-cell imaging	IL-2Rα	CD62L	1) Fast- or slow-cycling T-cell subsets after two to three divisions. 2) Forty percent of T produced both fast- and slow-subsets with difference in IL-2Rα expression.	(25)
Quezada et al. (2023)	scRNA-seq	Armstrong or C13 LCMV scRNA-seq and scATAC-seq	Enriched exhaustion-associated genes in C13 chronic infection		1) Two RNA-seq clusters and single ATAC-seq cluster in first-division Armstrong T cells. 2) Single RNA-seq cluster and three ATAC seq clusters in first-division C13 T cells. 3) Differentiation divergence between acute and chronic T-cell states already imprinted at first-division.	(43)
Gräbnitz et al. (2023)	Microscopy	CD8+ T cells activated by immobilized anti-CD3/CD28/ICAM1 or peptide-pulsed DCs Time-lapse microscopy	CD8 and CD62L–TCF1–	CD62L+TCF1+	1) CD8+ T cells silenced TCF1 after two to three divisions. 2) Strong TCR stimulation promoted asymmetric division. 3) Asymmetric divided cells formed colonies consisting of both effector (CD62L–TCF1–) and memory precursor cells (CD62L+TCF1+).	(24)

Depending on the system, the asymmetric division is usually observed in 50%–70% of T cells within the population. Why only half of the population divides asymmetrically is still an open question requiring further elucidation. Moreover, in the light of the recent study, asymmetric division may happen even less frequently than thought previously (45).

Although there are several established *in vivo* models to study the asymmetric division in T cells, it is necessary to address this phenomenon in other models including the cancer setting. It would be interesting to investigate its role during antitumor T-cell priming and immunotherapy treatment. Same is true for the asymmetric division markers as now T-cell division asymmetry is defined by arbitrary thresholds (e.g., levels of CD8 expression) chosen by different research groups. Indeed, several publications equate sorted high and low CD8/CD4 daughter cells with proximal and distal daughters, respectively. This is misleading as there might be a stochastic formation of high and low CD8/CD4 cell populations not related to the asymmetric division. This methodological gap is the reason why the biological significance of the asymmetric division in T cells still remains controversial (19, 31, 46). Deterministic universal division asymmetry markers are required to align the field and easily compare conclusions from different groups.

It is still unclear if memory T cells from the asymmetric division are superior to symmetrically divided memory T cells. To address this question single-cell lineage tracing experiments are required as routinely used FACS sorting of high or low expressing (CD8, IL-2Rα, and CD98) first-division T cells does not necessarily mean that they emerged from the asymmetric division. There is a need for a method that will precisely inhibit the asymmetric division without perturbing other essential signaling pathways in T cells. So far, aPKC or mTOR inhibition was used for that purpose. Yet, in addition to regulating the asymmetric division, aPKC and mTOR have a plethora of other functions in T cells, so aPKC/mROR inhibition or knockout cannot be treated as precise inhibitors of the asymmetric division (47). Finally, most of the work so far has focused on CD8+ T cells; hence, studies in other T-cell subsets including tissue-resident or regulatory T cells are also warranted. It will be interesting to see whether major histocompatibility complex (MHC) class I and MHC class II complexes influence ACD differently. In conclusion, the pivotal role of asymmetric division in T cells underscores its significance as a promising avenue for advancing immunotherapy strategies, offering new insights and opportunities to harness the immune system’s potential for targeted and effective treatments against various diseases.

## Author contributions

YK: Conceptualization, Writing – original draft. IG: Visualization, Writing – original draft. VC: Writing – review & editing. AK: Writing – review & editing. EB: Writing – review & editing.
